# New media, lifestyle transformation, and public health: unraveling the digital divide in China

**DOI:** 10.3389/fpubh.2025.1628076

**Published:** 2025-07-11

**Authors:** Ruize Ma, Tenglong Zheng, Mengxin Wang, Jiasheng Gu

**Affiliations:** ^1^School of Mental Health, Wenzhou Medical University, Wenzhou, China; ^2^Veterans Development Research Center of the Institute of Social Work and Development, Renmin University of China, Beijing, China; ^3^School of Social Research, Renmin University of China, Beijing, China

**Keywords:** public health, lifestyle, digital divide, new media use, lifestyle transformation

## Abstract

New media has profoundly reshaped everyday life in China, particularly influencing leisure lifestyles and raising new public health concerns. As the use of new media continues to rise, its impact on social behavior and well-being has become an urgent area of study. This study uses data from the 2018 Chinese General Social Survey (CGSS) and employs factor analysis and Oaxaca-Blinder decomposition to assess the impact of new media usage on lifestyle diversity across different social groups. The analysis focuses on various aspects of leisure activities and time use. Our findings show that increased use of new media promotes social leisure activities but leads to a reduction in participation in cultural and entertainment activities. Furthermore, lifestyle diversity is significantly lower among frequent new media users, with 50.7% of the disparity in lifestyle diversity attributed to the digital divide. This study suggests that while new media facilitates social interaction, it also fragments time use and may have negative consequences for mental well-being and social richness. To address these issues, policy responses should aim to reduce the digital divide and balance digital engagement with the need for diverse and healthy leisure activities, promoting both social connection and mental health.

## 1 Introduction

In recent years, with the improvement of socio-economic levels, public health issues caused by obesity have gained attention from government authorities. In 2025, China began fully implementing the “Weight Management Year” social policy, aiming to widely establish a supportive environment for weight management, significantly enhance public awareness and skills in weight management, promote healthier lifestyles, and gradually form a favorable situation where everyone participates and benefits from weight management, with improvements in abnormal weight conditions among certain populations. Among these efforts, lifestyle has become a key factor influencing public health.

It is well known that lifestyle is not merely a simple matter of individual subjective choice. Lifestyle refers to the behavioral patterns, consumption habits, leisure activities, and values that people exhibit in their daily lives (1–3). It not only reflects an individual’s personal choices but also mirrors the overall cultural atmosphere and economic development level of the society. In sociology, lifestyle is regarded as a symbol of social identity and status, and an important way for people to position and identify themselves in the society, which is of great significance for understanding social structure, social mobility, and social change. More importantly, lifestyle is closely intertwined with public health.

However, a background that cannot be ignored is that media consumption behaviors, such as watching television and binge-watching short dramas, have become one of the primary lifestyles for people today. With the rapid advancement of globalization and informatization, new media has been deeply integrated into people’s daily lives. Taking China as an example, the number of Chinese netizens has reached 1.051 billion as of June 2022, with an additional 19.19 million compared to December 2021. Moreover, the average weekly online time of netizens is 29.5 h, and the proportion of using mobile phones for internet access is as high as 99.6%.^1^ The popularity of new media not only changes people’s communication methods but also profoundly influences their lifestyles. Along with the prevalence of new media, people are not only keen on sharing their lives and opinions on the internet, but more importantly, the culture and values in online communities are also transmitted to people’s daily lives in a certain form, exerting a profound impact on people’s lifestyles.

In medical research, a healthy lifestyle (e.g., balanced diet, regular exercise, adequate sleep) directly impacts physical and mental well-being, while unhealthy lifestyles (e.g., sedentary behavior, excessive screen time, social isolation) may contribute to obesity, mental health issues, and an increase in chronic diseases (4). In daily life, how can we choose a rational lifestyle? Against the backdrop of the development and widespread adoption of new media technologies, particularly social media, fundamental changes have occurred in social interaction patterns and time allocation, which may influence individual health behaviors. Over the past few decades, China, as a nation undergoing modern transformation, has experienced rapid advancements in new media technologies alongside significant improvements in individual economic levels and internet usage. This raises the question: what impact does exposure to new media have on individuals’ lifestyles? Does this impact evolve with the advancement of information technology?

## 2 Literature review and research hypotheses

### 2.1 From traditional media to new media: concept and relevance

To better understand how new media reshapes lifestyle patterns, it is necessary to clarify how it differs from traditional media and why this distinction matters in the current research context. In this article, we adopt a tool-oriented perspective to conceptualize “new media.” From this viewpoint, new media is not merely an extension of technology but a medium ecology that redefines the production, dissemination, and consumption of information. Lev Manovich provides a clear definition in his work The Language of New Media. He argues that “media become new media” because the content of traditional media—such as graphics, moving images, sounds, shapes, spaces, and texts—can be translated into computer-accessible data through digitization (5). This translation process not only alters the form of media but also endows new media with characteristics of dynamism, interactivity, and modularity. For instance, digitized images can be broken down into pixel data, texts can be encoded into binary sequences, and sounds can be transformed into digital waveforms. These digital attributes grant new media a high degree of manipulability, reconfigurability, and cross-platform dissemination capabilities, setting it apart from traditional media.

In terms of specific forms, new media manifests in everyday life through various technological devices and platforms, including but not limited to smartphones, personal computers, laptops, tablets, the internet, and cloud-based applications and services. In contrast, traditional media encompass print newspapers, magazines, radio, television, and film (6). Traditional media typically rely on one-way communication, with fixed roles for content producers and consumers and a linear flow of information. New media, however, disrupts this model by enabling interactivity, user-generated content (UGC), and the proliferation of social media platforms, which grant users greater agency. For example, platforms such as Twitter, WeChat, or TikTok not only allow users to consume content but also enable real-time creation, sharing, and commenting, fostering a multidirectional and decentralized communication ecosystem.

### 2.2 New media and lifestyle

In sociology, lifestyle is broadly theorized as a process associated with marking differences—embedded in social order, delineating culture, and generating meaning. Indeed, this process can be regarded as a form of division of labor (7), through which individuals and collectives delineate “social differences derived from the use of resources rather than the mode of production” (8). This suggests that lifestyle represents a distinct modern form of status groups (8), a perspective fundamentally aligned with the cultural capital theory of the renowned sociologist Pierre Bourdieu.

Why do lifestyles undergo transformation? Many scholars attribute this primarily to changes in social structures (9). Identity is no longer fixed or stable, nor is it solely defined by an individual’s affiliation with traditional “status groups” (10), such as those conferred by nation-states, occupational classes, or family structures. Due to ongoing “detraditionalization,” individuals are increasingly liberated from traditional constraints, and identity is shaped within a context of multiple possibilities, emerging as a new concept with reflexive functions (3). Consequently, lifestyle transformation has become a new research direction in studies of social transformation. Particularly in the era of the network society, its decentralized and diversified structural characteristics have shifted the focus of lifestyles away from status systems toward shared interests and values.

Previous studies have explored the impact of new media on lifestyles from various perspectives, including the technology itself, its audiences, and the broader social environment. However, the definition and operationalization of lifestyle in these studies often appear overly simplistic. In reality, lifestyle is an extremely complex concept with rich research across different fields. It can refer to specific behavioral patterns and practices, such as consumption habits, leisure activities, and family practices, as well as to certain values and attitudes. Moreover, these dimensions vary and interconnect at both individual and collective levels (1, 2). Consequently, while prior research has demonstrated that new media induces changes in people’s lives, it remains unclear which specific aspects of lifestyle are affected and to what extent these changes can be attributed to new media.

In recent years, the development of new media has profoundly transformed lifestyles, prompting extensive and in-depth research in the field of sociology. First, new media has significantly altered social interaction patterns, enabled constant and ubiquitous communication and fostering the formation of new social networks (11). Additionally, new media has redefined individual and collective identity, allowing for the construction and reshaping of identities through “self-performance” in virtual environments (12). Furthermore, new media has had a profound impact on consumer culture, closely intertwining consumption behaviors with lifestyles and giving rise to a new culture of “consumption as lifestyle” (13).

Moreover, new media has reconfigured the boundaries between private and public life, blurring the distinction between these domains (14). This shift has influenced individuals’ perceptions of privacy and introduced new social norms (14). Additionally, the immediacy and high interactivity of new media have altered people’s perception and management of time, leading to the phenomenon of “fragmented time,” which disrupts traditional rhythms of life and work patterns (15). These studies not only deepen our understanding of modern lifestyle transformations but also provide new perspectives for future sociological research (16–18). Based on this, the following hypothesis is proposed:

*H1:* The use of new media will significantly affect people’s choice of lifestyles.

*H1a:* The higher the frequency of using new media, the more people tend to choose interest and hobby-based lifestyles.

*H1b:* The higher the frequency of using new media, the more people tend to choose social-based lifestyles.

*H1c:* The higher the frequency of using new media, the more people tend to choose cultural entertainment-based lifestyles.

With the development of information technology, the popularization of new media, and the rise of User-Generated Content (UCG), the “gatekeeper” effect of traditional media has gradually weakened. The information flow on new media platforms is no longer controlled by a few authoritative institutions but is produced and disseminated by many users themselves (19). This decentralized information dissemination model has brought about the diversity and pluralism of information, but it also comes with some new challenges. The dissemination of lifestyles in the new media environment has become more de-stratified, but there is a significant tension between this dissemination method and the stratified life in reality (14).

Specifically, users at the bottom of the social ladder on new media platforms often showcase themselves by imitating and learning the lifestyles of the upper class (20). This direct copying and learning, although accelerating the change of lifestyles, ignores the actual economic and social conditions of the bottom users. This seemingly “undifferentiated” dissemination covers up the underlying class differences, making lifestyles become superficial and homogeneous in media dissemination. The lifestyle displays on the new media platform are often beautified and screened, presenting an idealized and unrealistic state, and there is a significant tension between this state and the actual stratified life. Thus, the following hypothesis is proposed:

*H2:* In the process of lifestyle transformation, group disparities exist in both the explained and unexplained components associated with new media use.

## 3 Research methods

### 3.1 Research data

The data used in this paper are from the Chinese General Social Survey (CGSS) in 2010 and 2018, which is conducted by the Data Survey Center of Renmin University of China. CGSS is the earliest national, comprehensive and continuous academic survey project in China. Since 2003, CGSS has comprehensively collected data at multiple levels of society, community, family and individuals through scientific and systematic sampling, which is of great significance for summarizing the trends of social changes and exploring major social issues.

### 3.2 Variable settings

#### 3.2.1 Dependent variable

In the questionnaires of CGSS 2018 and CGSS 2010, the activities that the respondents engaged in during their leisure time were also surveyed. The interviewers asked the respondents about 12 kinds of leisure activities, including “watching TV or DVDs,” “going to the cinema,” “shopping,” “reading books/newspapers/magazines,” “participating in cultural activities,” “gathering with relatives,” “gathering with friends,” “enjoying music at home,” “doing physical exercises,” “watching sports games live,” “doing handicrafts,” and “surfing the Internet” (with options of “every day,” “several times a week,” “several times a month,” “several times a year or less,” and “never”).^2^

Considering that there are many sub-items in this question and they all belong to the secondary indicators under the lifestyle dimension, we conducted KMO and Bartlett’s sphericity test on the 12 activities. The results show that the *p*-value of Bartlett’s Sphericity Test is significant, indicating that the correlation coefficient matrix is not an identity matrix; Simultaneously, the KMO coefficient is 0.81, and the partial correlation coefficient is much smaller than the simple correlation coefficient, which is suitable for factor analysis. Using the maximum likelihood method, we performed spatial rotation based on factor analysis to obtain three factors.^3^ As shown in Table 1, the most frequently chosen activity by the public during their leisure time is “watching TV or DVDs,” followed by “surfing the Internet,” and the least chosen activity is “watching sports games live.” Thus, it can be drawn that with the development of modern society, surfing the Internet has become the main lifestyle, and new media has entered people’s lives.

**Table 1:** Factor analysis of lifestyles.

Item	Mean value	Standard deviation	Factor analysis			
			Hobby factor	Social factor	Cultural entertainment Factor	Communality
Watching TV or DVDs	2.027	2.947	−0.118	−0.005	**0.617**	0.606
Going to the cinema	4.529	3.197	0.318	0.133	**0.531**	0.599
Shopping	3.577	3.322	0.045	0.003	**0.549**	0.697
Reading Books/newspapers/magazines	4.071	3.587	0.381	0.267	**0.523**	0.510
Participating in cultural activities	4.719	3.480	0.337	0.338	0.414	0.601
Gathering with relatives	3.957	3.091	0.084	**0.798**	0.060	0.353
Gathering with friends	3.784	3.052	0.029	**0.836**	0.021	0.300
Enjoying music at home	3.615	3.774	0.395	**0.601**	0.206	0.441
Doing physical exercises	3.532	3.896	**0.654**	0.119	0.283	0.478
Watching sports games live	5.032	4.906	**0.865**	0.078	0.053	0.242
Doing handicrafts	4.851	4.746	**0.844**	0.084	0.018	0.280
Surfing the internet	2.928	3.564	0.420	0.193	0.431	0.600
Eigenvalue			2.6	1.96	1.72	6.28
Explained variance			21.71%	16.37%	14.37%	52.44%

The bold values indicate factor loadings greater than 0.5.

To further examine the role of new factors in the process of lifestyle changes, we calculated the factor scores and assigned weights according to the contribution ratio. We standardized each variable so that its mean is 0 and the standard deviation is basically 1, and weighted and summed them using the factor loading coefficients (F = ZC).^4^ Thus, a comprehensive lifestyle score is calculated for each sample. This score is a continuous variable that reflects the richness of the lifestyle and is used as the main dependent variable for our effect decomposition.^5^


$$\sum_{j=1}^{p}\left[x_{ij}-\mu_{1}\right)-\left(\alpha_{i1}\hat{f_{1}}+\alpha_{i2}\hat{f_{2}}+\cdots +\alpha_{im}\hat{f_{m}}\right){]}^{2}/\sigma_{i}^{2}$$

#### 3.2.2 Independent variables

This paper explores whether the lifestyle of the Chinese people is influenced by new media factors and whether the digital divide effect represented by new media exists in the disparities in the leisure lifestyle of the Chinese people. The main independent variables are media use and Internet use.

For the first independent variable, “media use,” we followed Hu and Zhuang (21) and Hu and Lin (22) approach to handling the “media usage” variable. We conducted a principal component analysis on the question “In the past year, how was your usage of six media types including newspapers, magazines, radio, television, the Internet (including mobile Internet access), and customized mobile phone messages?” in the CGSS 2018 questionnaire, and obtained two factors: “traditional media factor” and “new media factor.” Through Table 2, we can observe that “television” and “the Internet” are the most chosen media types by the public, while “magazines” have become the media with the lowest usage rate. The second independent variable is “Internet use.” We have extracted the use of the Internet as a main independent variable, which serves as a primary influencing factor for the decomposition of the lifestyle change effect.

**Table 2:** Factor analysis of media use.

Item	Mean Value	Standard Deviation	Factor Analysis		
			Traditional Media Factor	New media Factor	Communality
Newspapers	1.659	1.568	0.813	−0.192	0.302
Magazines	1.520	1.461	0.815	−0.175	0.306
Radio	1.599	1.552	0.756	−0.082	0.422
Television	3.551	1.848	0.115	0.312	0.889
Internet (including mobile Internet access)	2.938	2.661	0.220	0.746	0.395
Customized mobile phone messages	1.765	3.786	0.230	0.697	0.462
Eigenvalue			2.01	1.21	3.22
Explained variance			33.50%	20.20%	50.70%

Control variables include three categories. Firstly, regional indicators. Considering the impact of the regional economic level on the lifestyle, this paper selects the six major regions as the regional indicator variables. We form a new variable based on the regional economic level differences of the administrative regions where the respondents’ provinces are located, including North China, Northeast China, East China, Central South China, Southwest China, and Northwest China, with East China as the reference group. Secondly, this paper selects some indicators related to the socio-economic status as control variables, including years of education, logarithm of income, Party member (0 = no, 1 = yes), unit ownership system (0 = outside the state-owned system, 1 = within the state-owned system), household registration (0 = agricultural household registration, 1 = non-agricultural household registration), and occupational status (1 = unemployed, 2 = farmer, 3 = worker, 5 = clerical staff, 5 = self-employed, 6 = professional and technical personnel, 7 = managerial staff). Thirdly, considering the macro-environmental differences caused by the different social stages in which different birth cohorts are located, we add the birth cohort variable (1 = before 1960, 2 = after 1960, 3 = after 1970, 4 = after 1980, 5 = after 1990). Finally, this paper selects some basic demographic variables, including age, ethnicity (0 = minority, 1 = Han ethnicity), gender (0 = female, 1 = male), and current marital status (0 = unmarried, 1 = married) as control variables (see Table 3).

**Table 3:** OLS regression model of influencing factors of lifestyle.

Variables	Model 1	Model 2	Model 3	Model 4
	Hobby factor	Social factor	Entertainment factor	Lifestyle richness
Traditional media factor	−0.05	−0.05^*^	0.05^*^	−0.02
	(−1.52)	(−2.36)	(−2.23)	(−1.47)
New media factor	−0.01	−0.04^***^	0.02^*^	−0.01
	(−0.43)	(−3.98)	(−2.08)	(−1.37)
Logarithm of income	0.00	0.00	0.00	0.00
	(−0.21)	(−0.05)	(−0.03)	(−0.18)
Gender	−0.02	−0.01	0.07	0.01
	(−0.17)	(−0.13)	(−1.08)	(−0.22)
Age	0.02	0.05	0.01	0.03
	(−0.47)	(−1.66)	(−0.20)	(−1.22)
Square of age	0.00	0.00	0.00	0.00
	(−0.59)	(−1.54)	(−0.50)	(−1.00)
Ethnicity	0.20	0.05	−0.04	0.08
	(−1.12)	(−0.38)	(−0.34)	(−0.97)
Household registration	−0.31^**^	−0.19^*^	−0.38^***^	−0.29^***^
	(−2.62)	(−2.13)	(−4.47)	(−4.85)
Years of education	−0.01	−0.03^**^	−0.07^***^	−0.04^***^
	(−1.00)	(−3.21)	(−7.23)	(−5.06)
Marital status	0.01	0.06	−0.19^*^	−0.03
	(0.00)	(−0.66)	(−2.17)	(−0.53)
Party member status	−0.09	0.14	−0.03	0.00
	(−0.57)	(−1.26)	(−0.25)	(−0.01)
Unit ownership system	−0.15	0.16	−0.18	−0.06
	(−1.13)	(−1.63)	(−1.94)	(−0.95)
Generation (Ref: Born before 1960)				
1960s	−0.32	−0.10	0.26^*^	−0.09
	(−1.72)	(−0.75)	(−1.96)	(−1.00)
1970s	−0.29	0.16	0.24	0.00
	(−0.99)	(−0.75)	(−1.17)	(−0.02)
1980s	−0.54	0.19	0.43	−0.05
	(−1.17)	(−0.57)	(−1.32)	(−0.20)
1990s	−0.61	0.25	0.49	−0.04
	(−0.91)	(−0.52)	(−1.02)	(−0.12)
Occupation (Ref: Unemployed)				
Farmer	0.22	0.06	0.37^*^	0.21^*^
	(−1.04)	(−0.37)	(−2.49)	(−1.99)
Worker	0.33	−0.24	0.13	0.10
	(−1.72)	(−1.72)	(−0.96)	(−1.01)
Clerical staff	0.24	−0.52^**^	−0.05	−0.08
	(−0.99)	(−2.93)	(−0.31)	(−0.64)
Professional/technical personnel	0.06	−0.46^**^	0.12	−0.09
	(−0.27)	(−2.86)	(−0.76)	(−0.78)
Managerial staff	0.89^*^	−0.70^*^	−0.25	0.08
	(−2.37)	(−2.55)	(−0.93)	(−0.44)
Six major regions (Ref: North China)				
Northeast China	0.16	−0.43^**^	0.12	−0.03
	(−0.86)	(−3.13)	(−0.88)	(−0.37)
East China	−0.02	−0.32^***^	0.08	−0.09
	(−0.18)	(−3.44)	(−0.88)	(−1.37)
Central-South China	−0.11	−0.43^***^	0.08	−0.16^*^
	(−0.74)	(−4.04)	(−0.75)	(−2.16)
Southwest China	0.35^*^	−0.32^**^	0.26^*^	0.11
	(−2.06)	(−2.65)	(−2.21)	(−1.35)
Northwest China	0.08	−0.48^***^	0.14	−0.08
	(−0.44)	(−3.64)	(−1.10)	(−0.87)
_cons	4.02^**^	3.01^**^	3.58^***^	3.54^***^
	−2.78	−2.85	−3.49	−4.93
*N*	10,384	10,384	10,384	10,384
adj. *R*^2^	0.01	0.02	0.04	0.04

*t* statistics in parentheses +*p* < 0.1, ^*^p < 0.05, ^**^p < 0.01, ^***^p < 0.001.

## 4 Research findings

### 4.1 Analysis of influencing factors of lifestyle

Considering that in the operationalization part of the previous text, the “hobby factor,” “social factor” and “cultural entertainment factor” of lifestyle have been calculated and scored, forming continuous numerical variables. Therefore, we choose to use a linear regression model for the multivariate analysis of influencing factors.

The data results of Model 1 show that in the lifestyle of the Chinese people, neither traditional media nor new media have had a significant impact on the lifestyle type in which the people choose hobbies as the main factor. Thus, H1a is not verified. However, other significant variables suggest that the Chinese people will increasingly reject a lifestyle related to hobbies as they get older. But after a certain age, the impact of age will show an upward trend. The impact of age on a hobby-based lifestyle is U-shaped. Secondly, urban residents prefer a lifestyle related to hobbies more than rural residents, which is closely related to modernization. Cities provide the public with more activity options related to hobbies.

The data results of Model 2 show that traditional media and new media have a significant impact on a social-related lifestyle. The more frequently people use traditional media or new media, the more they will choose a social-based lifestyle, and the impact of new media is greater than that of traditional media. Thus, H1b is verified. It can be drawn that new media has changed people’s way of communication, triggering a positive social effect. People’s use of new media such as mobile phones and the Internet has significantly increased the possibility of social interaction. Simultaneously, other significant variables suggest that the years of education, occupation, household registration, occupation, generation and the region where they are located will significantly affect the choice of social-based lifestyle. As the years of education increase, people are fonder of a social-based lifestyle. The same explanation applies to the improvement of occupational status, urban residents, younger generations and economically underdeveloped areas. These group characteristics all increase the possibility of choosing a social-based lifestyle.

The data results of Model 3 suggest that both traditional media and new media will significantly affect the choice of a lifestyle related to cultural entertainment. The more frequently people use traditional media or new media, the less they will choose a lifestyle related to cultural entertainment. Moreover, the impact of traditional media is greater than that of new media, which may be because the increase in the use of the two media will reduce leisure time, so people are less likely to choose a lifestyle related to cultural entertainment such as going to the cinema and shopping. Thus, H1c is verified. From other significant variables, urban residents are fonder of a cultural entertainment-based lifestyle. The same explanation applies to the improvement of the years of education, married people, and people within the state-owned system. These group characteristics all increase the choice of a lifestyle related to cultural entertainment.

In Model 4, we calculated the factor scores and assigned weights according to the contribution ratio to calculate a comprehensive lifestyle score for each sample. This score is a continuous variable that reflects the richness of the lifestyle. The lower the value, the stronger the richness of the lifestyle. Since it is a continuous variable, the OLS multiple regression model is also adopted. The data results show that neither traditional media nor new media have a significant impact on the richness of the lifestyle. The choice of lifestyle is still significantly related to the traditional factors such as age, household registration, years of education, and occupational status, which is also in line with our common sense.

### 4.2 Oaxaca-blinder analysis of lifestyle richness

To further understand the exact role that the advancement of new media technology has played in the transformation of lifestyles, we used the data from 2010 and 2018 to form a panel dataset, and selected the “Oaxaca-Blinder” decomposition method to analyze whether there is a digital divide effect brought by new media in individuals’ choice of lifestyle. The “Oaxaca-Blinder” decomposition method is a classic approach to decomposing the mean wage disparity. It was almost simultaneously proposed by Ronald Oaxaca and Alan S. Blinder and is widely employed in studies in economics and sociology. Then we use whether to use the Internet (including surfing the Internet) as the defining variable for the new media user group and the non-new media user group. In the CGSS questionnaire, respondents were asked about their use of the Internet (including surfing the Internet). Respondents were required to choose from five options based on their own situations: “Never,” “Rarely,” “Sometimes,” “Often,” and “Very frequently.” We processed the variables, classifying those who answered “Never” as the non-new media user group, and the rest as the new media user group. Thus, a new variable, “Whether it is a new media user group or not,” was generated.

As is well known, there are diverse reasons for the new media user group and the non-user group to choose their lifestyles. Moreover, the use of new media often requires corresponding technical equipment (such as computers and smart phones) and certain expenses (such as network fees and data traffic fees). In other words, the underlying logic is that the differences in the socio-economic status between the new media user group and the non-user group may directly affect the choice of lifestyle. For example, suppose the non-new media user group has a lower level of education, then the difference in lifestyle due to the use of new media may reflect the difference in educational level between the groups. Therefore, if we want to explore whether there is a new media digital divide effect in the choice of people’s lifestyle, we need to construct a counterfactual group, that is, to construct “new media users who are regarded as non-new media users.” If there is no new media digital divide effect in the choice of lifestyle, then the lifestyle richness of this counterfactual group should not be significantly different from that of the new media user group.


(1) lifestyle=Xβ+ε

In Equation 1, the dependent variable *lifestyle* represents lifestyle richness, while X denotes the vector of structural factors, including years of education, gender, income, age, Party member status, occupational status, unit ownership status, ethnicity, marital status, household registration, generation and economic region. *β* is the corresponding coefficient estimate, and *ε* is the error term. Based on this, the lifestyle difference between the new media user group and the non-new media user group is decomposed into an explainable part and an unexplainable part. This difference in the richness of the lifestyle stems from the lifestyle differences brought about by various structural factors. Here, the lifestyle richness of the new media user and the non-new media user is respectively:


(2) $$\bar{lifestyle_{u}}=\bar{X_{u}}\;\hat{\beta_{u}}$$

(3) $$\bar{lifestyle_{m}}=\bar{X_{m}}\;\hat{\beta_{m}}$$

Where the subscript u represents the non-new media user group, m represents the media user group, and lifestyle is the lifestyle richness obtained through the factor comprehensive score calculation formula mentioned earlier. $X_{u}$ is the mean value of the explanatory variables in the non-new media user group sample, and $\beta_{u}$ is the regression coefficient of Equation 2. The same explanation applies to the meaning of each variable in Equation 3. Thus, the lifestyle richness difference between the new media user group and the non-new media user group can be decomposed as:


(4) $$\bar{lifestyle_{u}}-\bar{lifestyle_{m}}=\left(\bar{X_{u}}-\bar{X_{m}}\right)\hat{\beta_{m}}+\left(\hat{\beta_{u}}-\hat{\beta_{m}}\right)\bar{X_{u}}$$

The first part on the right side of Equation 4 is the explainable part (characteristic effect), and the second part is the unexplainable part (coefficient effect). We regard the coefficient effect as the digital divide effect between new media and traditional media.

The Oaxaca-Blinder decomposition results (Table 4) indicate that the average lifestyle diversity score for the non-new media user group is 4.467, while that for the new media user group is 3.608, resulting in a gap of 0.859 (approximately a 23.8% difference). Of this gap, approximately 49.3% is attributed to differences in demographic characteristics between the groups (e.g., household registration, age, education level). The remaining 50.7% is due to the “unexplained portion,” which reflects varying returns to new media usage among individuals with similar characteristics, manifesting as the “new media digital divide effect.”

**Table 4:** Oaxaca-blinder decomposition of lifestyle richness.

Decomposition components	Lifestyle richness	
Overall		
Non-new media user group	4.467^***^	97.71
New media user group	3.608^***^	163.04
Total variation in lifestyle richness	0.858^***^	16.89
Explainable variation	0.424^***^	9.72
Unexplainable variation	0.435^***^	6.53
Contribution of structural factor differences		
Generation	0.008	1.54
Gender	−0.003	−1.51
Age	0.090^*^	2.41
Ethnicity	−0.001	−0.23
Household registration	0.081^***^	5.82
Years of education	0.232^***^	5.80
Marital status	−0.004	−1.77
Party member	0.006	1.60
Occupational status	0.014	0.9
Region	0.002	0.58
Income	−0.002	−0.14
Differences in contribution of new media digital divide effect		
Generation	−0.001	−0.35
Gender	−0.054	−1.15
Age	−0.510	−1.65
Ethnicity	0.130	0.76
Household registration	0.031	0.81
Years of education	0.122	1.54
Marital status	−0.190^*^	−1.99
Party member	0.032^*^	1.97
occupational status	−0.209	−1.55
Region	−0.100	−0.87
Income	0.054	0.54
Sample size	10,452	/

*t* statistics in parentheses +*p* < 0.1, ^*^p < 0.05, ^**^p < 0.01, ^***^p < 0.001.

Specifically, the structural difference in years of education has the most significant explanatory power for the group disparity, while differences in Communist Party membership and marital status are also notable. Therefore, the impact of new media usage on lifestyle diversity is not homogeneous but is shaped by the combined effects of structural differences and variations in the marginal returns to technology use.

Specifically, the lifestyle diversity gap can be decomposed into two components. In the explained portion, differences in structural factors (e.g., gender, age, ethnicity, household registration, years of education, Communist Party membership, occupational status, and geographic region) between new media users and non-users result in disparities in lifestyle diversity, even when a counterfactual group is constructed. More precisely, 49.3% of the lifestyle diversity gap among Chinese citizens can be attributed to these structural factor differences. In other words, structural factors such as gender, age, and years of education, which are correlated with lifestyle diversity, account for 49.3% of the gap. Among these, changes in years of education (including variations in structural factor coefficients and the new media digital divide effect coefficients) have the strongest explanatory power for changes in lifestyle diversity.

In other words, 49.4% of the difference in lifestyle diversity between new media users and non-users is attributed to the explained component, primarily driven by structural disparities in education, hukou status, and age, underscoring the role of social stratification in shaping lifestyle diversity. Education level contributes the most, indicating that higher-educated individuals are more likely to achieve diverse lifestyles through resource access. Urban–rural hukou disparities reflect the benefits urban residents gain from superior infrastructure. Age differences suggest that younger individuals, despite frequent new media use, do not necessarily translate this into richer lifestyles. The unexplained component captures the potential effects of new media use, with marital status and party membership showing significant roles: new media use among married individuals may reduce offline interactions, leading to less diverse lifestyles, while party members may benefit from enhanced information access or social engagement. Other factors, such as age and occupational status, exhibit negative but non-significant effects. These findings indicate that new media, as a technological tool, has a dual impact on lifestyle, modulated by usage patterns and social roles.

Thus, H2 is verified. We can draw a basic conclusion that with the progress of Chinese society and the improvement of modernization, the characteristics of the network society have become prominent. The use of the Internet has been integrated into people’s lives and has changed people’s lifestyles. When we connect the society through new media including the Internet, a “disembedding” life has emerged. New ways of socializing and obtaining information that are free from spatial restrictions are gradually changing our choice of lifestyles.

## 5 Conclusion and discussion

Based on the current analysis and results, this study primarily examines the mechanisms through which new media usage influences lifestyle choices and confirms the existence of a new media digital divide. The model results suggest potential mechanisms and effects of new media in areas such as facilitating social interaction, information acquisition, and interest expansion, personalizing cultural and entertainment experiences, digital alienation, and the interaction between regional economic development and new media usage.

First, the study indicates that new media significantly enhances social leisure lifestyles but reduces participation in cultural and entertainment leisure activities. From a traditional perspective, the advancement and development of new media appear to comprehensively enrich daily life, enabling the discovery of interests and hobbies, the facilitation of social interactions, and the selection of leisure and entertainment options through new media platforms (23, 24). However, our analysis reveals that new media does not positively impact leisure lifestyles related to interests, hobbies, or cultural entertainment. This divergence can be attributed to the fundamental differences in time structure, social interaction settings, and psychological fulfillment between various types of leisure activities. Social-oriented activities are often instant, interactive, and require low thresholds of participation, aligning closely with the fragmented and accessible nature of new media. In contrast, traditional cultural and entertainment activities require prolonged engagement and deep experiences. The fast-paced and immediate entertainment options offered by new media (e.g., short videos, social platforms) foster alienation and resistance toward traditional entertainment, particularly when virtual social interactions already fulfill social needs.

Secondly, exposure to new media has increased people’s preference for spending leisure time gathering with relatives and friends, indicating that new media has strengthened social connections among Chinese citizens. The Chinese social network, rooted in kinship, professional, and geographic ties, has been effectively promoted and advanced within the realm of new media. China has historically been a civilization that values etiquette and filial piety. Despite the disruptions to traditional culture caused by industrialization and modernization, which have led to perceptions of growing interpersonal alienation, new media has comprehensively extended the continuity of traditional culture by enhancing social connections between individuals.

Further analysis reveals that, contrary to traditional assumptions, new media does not enhance lifestyle diversity by providing access to more activity scenarios. On the contrary, compared to individuals who have not engaged with new media, new media usage is associated with a decline in lifestyle diversity. The reason lies in the “disembedding” characteristic of new media, which, to some extent, reduces opportunities for participation in real-world, physical social and entertainment activities, resulting in a “flattening” effect on social life. While new media exposes individuals to a wealth of information and lifestyles, thereby diversifying their information sources, it also triggers negative effects such as alienation, resistance, and even disorientation.

In traditional understanding, new media was expected to enhance technological convenience and elevate lifestyles. However, the over-digitization of information and reliance on algorithms have led to a decline in individuals’ control over information. Information is no longer merely a medium of content transmission but is shaped by multiple ideologies and algorithmic “processing.” This information alienation enables groups that have not engaged with new media to maintain relatively “authentic” lifestyles. Moreover, lifestyles detached from technology exhibit a heightened contrast, further accentuating this divergence.

The black box is a concept in cybernetics. After eliminating the relevant factor of “if using new media, there is no time to carry out other activities” in the traditional perception, we have verified from the models the impact that new media itself brings on objective individuals. Contrary to the assumption, compared with those who have never been exposed to this technology of new media, new media reduces the richness of people’s lives by 23.8%. The reasons are as follows:

Firstly, the “disembedding” lifestyle exerts a leveling effect on the differences in people’s lives. As an emerging technology carrier, the “disembedding” function of new media has a leveling effect on the class differences in lifestyles. People can access entertainment and consumption information and living habits that they would not access in traditional life through the new media carrier, and this information can cause negative effects such as resistance and disorientation.

Secondly, there is digital alienation in the digitization of the lifestyle. In the traditional perception, new media itself will bring technological dependence, digitization of entertainment methods, and digitization of the lifestyle, while the digital economy will provide a convenient life for the Chinese people. However, we believe that the phenomenon of digital alienation has already occurred. The theory of alienation originated from Hegel and developed by Marx is an important thought for us to examine the object. When the development of the object is embedded with symbols of other wills, the object will deviate from itself and thus produce alienation. When the diverse and complex information is transmitted to individuals through new media, the processing and reproduction of information are embedded with various consciousness. In other words, for individuals, information is no longer simply information, but a processed entity embedded with complex multi-party consciousness. Thus, among those groups that have not been exposed to new media, the phenomenon of being disconnected from technology but having an enhanced lifestyle is manifested.

As mentioned earlier, the rapid development of new media has provided more choices and better support for meeting people’s needs for a better life on a larger scale. Against the backdrop of promoting a society of common prosperity, the Chinese government has introduced a series of policies, such as nationwide reading campaigns and fitness programs, to enrich residents’ leisure time. As a carrier of internet technology, new media has further provided Chinese citizens with numerous online leisure and entertainment options, such as virtual museums, online exhibition halls, and digital fitness centers. Governing lifestyles is an effective approach for the government to protect public health.

In summary, this study not only elucidates the multidimensional impacts of new media on lifestyles but also provides new perspectives for understanding its effects on social structures, social interaction patterns, and choices of cultural and entertainment activities. Future research should focus on optimizing the use of new media to mitigate its negative effects, promote healthier and more balanced lifestyle choices, and provide a theoretical basis for government policies on public health in the digital era.

Nonetheless, this study has certain limitations. While it focuses on the impact of new media on lifestyle transformation, it does not directly examine the substantive health outcomes of such lifestyle changes. Since lifestyle itself is not equivalent to public health, the extent to which media-induced lifestyle transformations translate into actual health behaviors or psychological well-being remains to be empirically tested. This limitation opens up important avenues for future research, particularly in the intersection of digital sociology and public health studies.

## Data Availability

Publicly available datasets were analyzed in this study. This data can be found here: http://www.cnsda.org/.

## Footnotes

^1^Data Source: Global and Regional ICT Data. ITU. (2021). ITU World Telecommunication/ICT Indicators Database. Retrieved from https://www.itu.int/en/ITU-D/Statistics/Pages/stat/default.aspx.

^2^Assign values from 1 to 5 respectively, with 1 indicating “every day” and 5 indicating “never.”

^3^We have conducted the same processing of the CGSS 2010 data for the lifestyle question. The KMO coefficient is 0.79, and the *p*-value of Bartlett test is significant. Based on the principal component analysis, a spatial rotation is performed to obtain two factors.

^4^Z is the standardized data, and C is the factor score coefficient matrix.

^5^The $\hat{f_{1}}\cdots \hat{f_{m}}$ that minimizes this equation is the factor score of corresponding individual case.
