# Correction: New media, lifestyle transformation, and public health: unraveling the digital divide in China

**DOI:** 10.3389/fpubh.2025.1664479

**Published:** 2025-08-08

**Authors:** Ruize Ma, Tenglong Zheng, Mengxin Wang, Jiasheng Gu

**Affiliations:** ^1^School of Mental Health, Wenzhou Medical University, Wenzhou, China; ^2^Veterans Development Research Center of the Institute of Social Work and Development, Renmin University of China, Beijing, China; ^3^School of Social Research, Renmin University of China, Beijing, China

**Keywords:** public health, lifestyle, digital divide, new media use, lifestyle transformation

In the published article, there was an error regarding the affiliation(s) for [Mengxin Wang and Jiasheng Gu]. As well as having affiliation(s) [1], they should also have [*School of Social Research, Renmin University of China, Beijing, China*].

The original version of this article has been updated.

